# Cable-routing geometry as a design variable for multiplanar hip assistance in a single-actuator soft exosuit

**DOI:** 10.1371/journal.pone.0359731

**Published:** 2026-10-29

**Authors:** Junhyeong Kwon, Sejun Park, Harry George Driscoll, Giuk Lee

**Affiliations:** 1 Department of Mechanical Engineering, Chung-Ang University, Seoul, Republic of Korea; 2 HUROTICS Inc., Seoul, Republic of Korea; Indian Institute of Technology Patna, INDIA

## Abstract

Cable-driven soft exosuits for gait assistance are predominantly sagittal-plane devices, yet pathological gait involves coordinated deviations across all three anatomical planes. This study experimentally evaluated whether cable-routing geometry alone, within a single-actuator hip exosuit, can produce measurable multiplanar hip effects during walking. Ten healthy, exosuit-experienced male adults walked on a treadmill under five conditions: one matched unpowered control and four powered configurations that paired single-plane and multi-plane cable routing with hip-flexion and hip-extension assistance. Three-dimensional hip kinematics were compared across conditions, and exosuit-generated hip torques were estimated from subject-specific musculoskeletal models. Multi-plane routing produced phase-dependent kinematic changes concentrated in the transverse plane. Relative to unassisted walking, multi-plane flexion assistance increased hip internal rotation (3.6°, p = 0.0002), multi-plane extension assistance increased hip external rotation (1.8°, p = 0.0095), and single-plane flexion assistance produced a smaller but significant increase in external rotation (1.1°, p = 0.0463). Frontal-plane differences were not significant for any comparison with unassisted walking, so the hypothesized larger frontal-plane deviations under multi-plane routing were not supported. Multi-plane routing also produced greater sagittal-plane hip excursion than single-plane routing. Overall gait-trajectory similarity between single-plane and multi-plane routing was comparable in most comparisons and differed significantly only in the hip frontal plane during extension assistance. The estimated torques indicated that multi-plane routing introduced larger frontal- and transverse-plane components while the sagittal-plane component remained dominant. These findings show that cable-routing geometry alone can shape phase-localized hip kinematics, reaching significance in the transverse plane, without adding actuators. The effect magnitudes were small and near the measurement uncertainty of the capture system. The all-male, exosuit-experienced sample limits generalizability, so relevance to clinical populations remains to be tested. Cable-routing geometry is therefore proposed as a candidate rather than an established design variable for multi-dimensional assistance in soft exosuits.

## Introduction

Lower-limb assistive devices, such as exoskeletons, have been developed to assist human walking. While many exoskeletons are designed with rigid structures, recent work has increasingly focused on cable-driven soft exoskeletons, or exosuits [1–4]. These exosuits are characterized by lightweight, flexible materials that allow them to provide assistive joint torques while minimally restricting natural gait biomechanics [5]. These wearable robots have been shown to improve lower-extremity gait performance and biomechanics across diverse populations, ranging from older adults [6,7] to individuals with pathological gait caused by neuromuscular impairments [8–10].

Pathological gait associated with neuromuscular impairments such as stroke [5] or cerebral palsy [7] involves complex three-dimensional (3-D) movement patterns that extend beyond abnormalities in a single joint or plane. For example, Kim et al. showed that individuals with chronic stroke use diverse gait strategies, including a compensatory hip abductor moment in the frontal plane to aid swing-phase clearance and a knee flexor moment pattern in the sagittal plane that is often associated with a knee hyperextension thrust [6]. Similarly, children with cerebral palsy commonly exhibit dynamic hip deviations in the transverse [10] and frontal [11] planes during walking. These studies indicate that biomechanical abnormalities in lower-limb joints extend beyond flexion-extension deficits alone. Accordingly, gait-assistive exosuit design should consider not only assistance magnitude and timing but also how the force line is routed relative to the joint so that assistance can interact with frontal- and transverse-plane mechanics in a controlled manner. Despite the 3-D nature of pathological gait, most cable-driven exosuits for gait rehabilitation remain largely sagittal-plane dominant, typically providing single-plane, single-degree-of-freedom (DOF) assistance at the hip [9,12,13] or ankle [14,15]. Consequently, existing designs often neglect the influence of cable-path geometry on off-sagittal torque generation and joint coordination during walking.

More recently, cable routing has gained attention as an important design factor in soft exosuits. Simulation and experimental studies have suggested that cable-path geometry can influence biomechanical outcomes during walking. Prasad et al. [16] proposed a simulation-based cable-routing framework showing that routing geometry can alter joint torque generation across the sagittal and frontal planes. However, their framework was limited to a simplified 3-DOF model involving sagittal- and frontal-plane motion, excluded hip transverse-plane rotation, and evaluated routing performance through trajectory-tracking simulations rather than experimental observations of human walking. Accordingly, it did not determine whether routing geometry alone can elicit measurable multiplanar gait alterations in humans under actual walking conditions. Lambranzi et al. [17] demonstrated that modifying anchor-point placement in a hip-flexion assistive exosuit can alter energetics and lower-limb biomechanics, providing experimental evidence that exosuit geometry influences biomechanical responses. However, their analysis focused primarily on overall performance differences across configurations without explicitly examining whether routing geometry can be used to deliberately shape localized 3-D joint kinematics. In particular, a direct comparison between single-plane and multi-plane routing strategies and their associated biomechanical effects is still lacking. Likewise, Zhang et al. [18] demonstrated that, at the ankle, the position of the exosuit force line relative to the subtalar joint axes substantially affected inversion, adduction, and kinematic similarity to active plantarflexion. Taken together, these studies suggest that routing geometry matters. However, it remains unclear whether a single-actuator cable-driven soft exosuit can produce functionally meaningful multiplanar biomechanical effects solely through cable-routing geometry, rather than through an additional actuator or an explicitly independent frontal/transverse control source.

To address this gap, we experimentally examined whether cable-routing geometry alone can be used to shape multiplanar biomechanical effects in a hip-assisting soft exosuit. In doing so, the present study provides direct experimental evidence that routing geometry alone can induce measurable off-sagittal hip effects during walking. Specifically, we compared monoarticular single-plane (SG) routing and biarticular multi-plane (MT) routing under matched flexion- and extension-assistance conditions. The MT configurations used an oblique pathway terminating near the proximal tibia, whereas the SG configurations used a distal femoral pathway intended to preserve a predominantly sagittal-plane line of action. This design allowed routing geometry to be isolated as the primary variable of interest while maintaining identical sagittal-plane actuation across conditions. The primary objective of this study was to provide direct experimental evidence that cable-routing geometry alone, within a single-actuator exosuit, can produce measurable multiplanar hip effects during walking. We hypothesized that (H1) MT assistance would induce larger frontal- and transverse-plane kinematic deviations than SG routing, given the oblique cable trajectory designed to generate multiplanar hip torques. We further hypothesized that (H2) these localized off-sagittal effects would occur without causing larger deviations in overall gait-trajectory similarity than SG routing.

## Materials and methods

### Exosuit design and control

To provide walking assistance, we used the H-Medi (HUROTICS, Inc., Seoul, Republic of Korea), a cable-driven soft exosuit [19], as shown in Fig 1. The system consists of a main module weighing approximately 3.5 kg, which houses two actuation motors, a battery, and a controller, and a textile garment. The actuation motors transmit assistive forces through Bowden cables routed through a pulley mechanism. The exosuit delivers assistive forces through anchor points secured to a garment consisting of a waist belt, thigh pieces, and knee braces. The waist belt functions as the proximal anchor, whereas the distal cable ends were attached to either the thigh pieces or the knee braces, depending on the assistance configuration. A commercial knee brace (ZK PROJECT, Nippon Sigmax Co., Ltd., Tokyo, Japan) was customized by modifying its anchor points to create multiplanar cable pathways. This garment structure allows the desired cable pathways to be routed while minimizing interference with natural movement.

**Fig 1:**
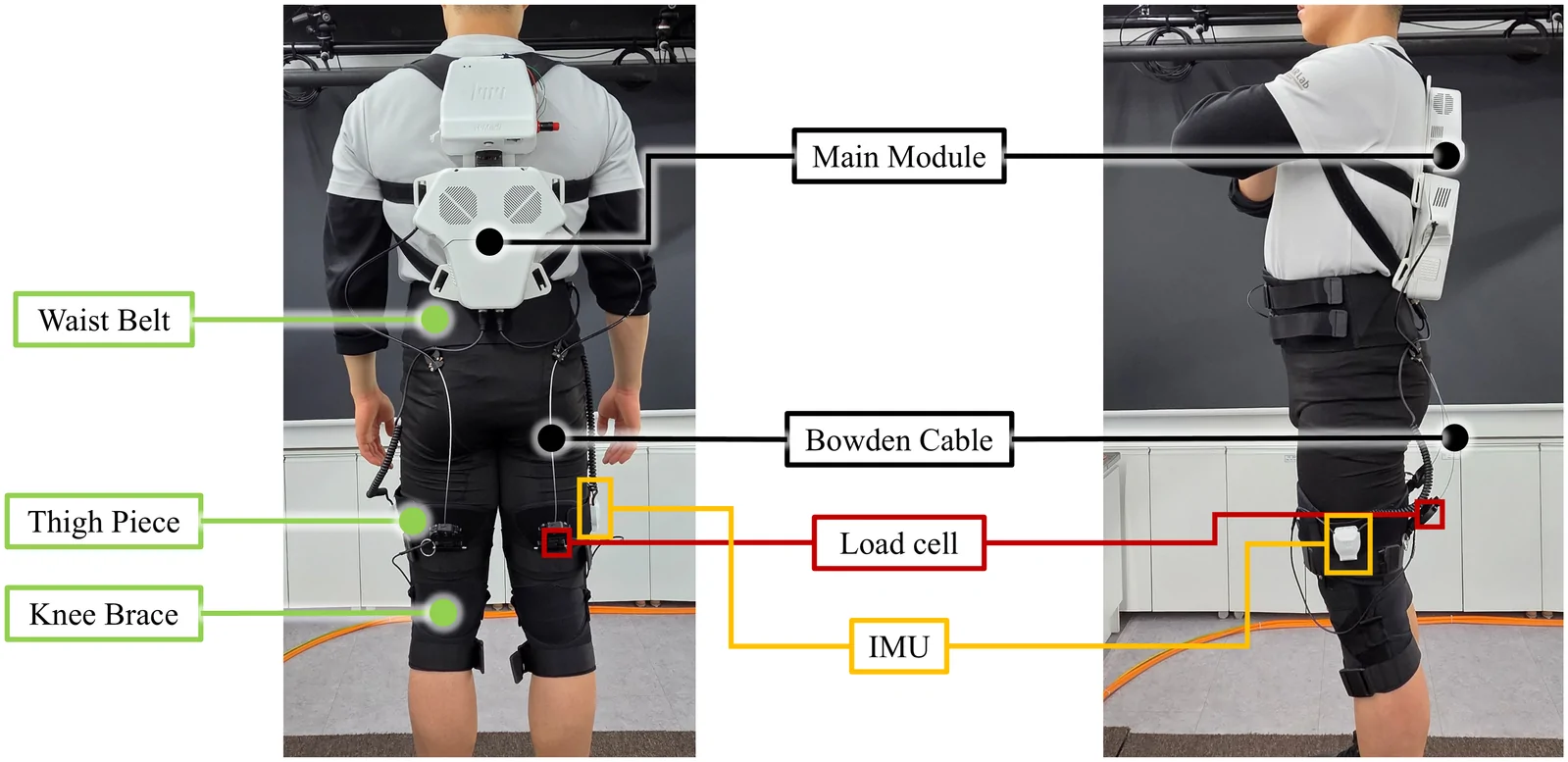
Exosuit hardware and its components. The system is composed of following component: robot main units (black boxes), pieces and braces (green boxes), sensors (red and yellow boxes). This photograph published under a CC BY 4.0 license, with written permission from the individual depicted.

To estimate the gait cycle in real time, inertial measurement unit (IMU) sensors were mounted on the thigh pieces. The gait cycle was identified from hip joint events, specifically maximum hip extension (MHE) and maximum hip flexion (MHF). These IMU signals were used solely to time the assistance profile. All reported joint kinematics were derived exclusively from the markerless OpenCap system described below, and the two data streams were not fused. In addition, load cells were installed at the distal ends of the cables to measure the assistive force delivered to the user. The assistance force profile applied in this study followed a half-sine wave defined by three gait-timing parameters, onset, peak, and offset. These parameters denote the timing, as a percentage of the gait cycle, of the assistive-force profile itself rather than of joint torque or joint angle. We derived these values from previous optimization studies in healthy adults. For hip flexion assistance, where MHE was defined as the start of the gait cycle (0%), the onset, peak, and offset occurred at 0%, 18%, and 38% of the gait cycle, respectively [20]. For hip extension assistance, where MHF was defined as 0%, the onset, peak, and offset occurred at 0%, 35%, and 52%, respectively [21]. Both force profiles are illustrated on a common normalized gait cycle in Fig 2, with MHF and MHE placed at 0% and 60%, respectively, so that each profile is displayed relative to the event that defined its timing. Although these optimized force profiles were originally developed for single-plane assistance and their suitability for multi-plane assistance remains unverified, we controlled the assistance profile to primarily evaluate the effects of different cable pathways. Therefore, the force profile was selected solely on the basis of the assistance action (extension or flexion), regardless of the movement plane. This design choice allowed an isolated evaluation of routing geometry, but it may have underestimated the kinematic effects achievable with timing profiles optimized specifically for multiplanar assistance.

**Fig 2:**
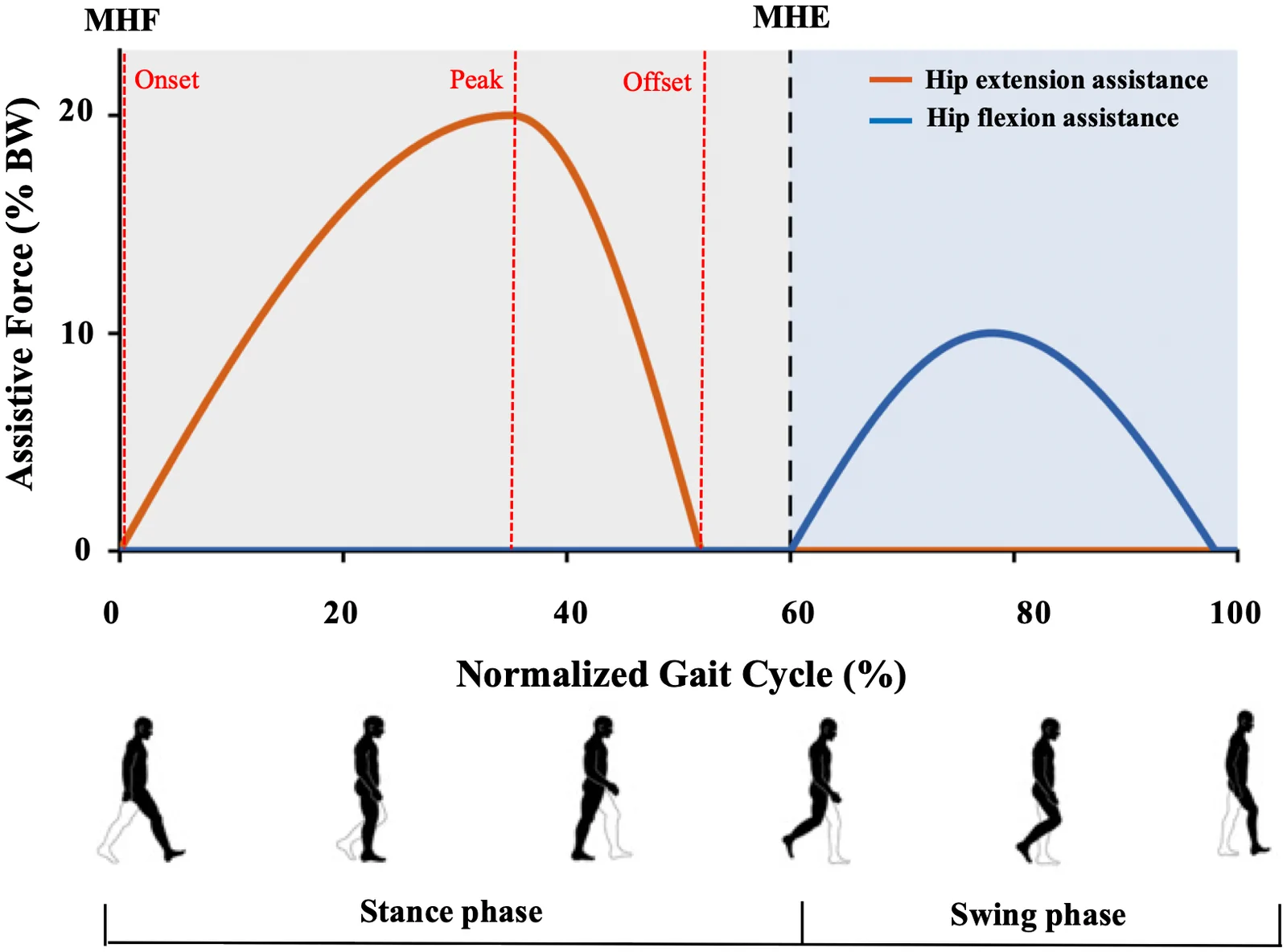
Desired half-sine assistive-force profiles for hip flexion and hip extension across the gait cycle. Each assistance mode followed an asymmetric half-sine profile defined by three gait-timing parameters (onset, peak, and offset). Hip extension assistance (orange) acted during the stance phase, with onset, peak, and offset at 0%, 35%, and 52% of the gait cycle; hip flexion assistance (blue) acted during the swing phase, with onset, peak, and offset at 0%, 18%, and 38%. Both profiles are shown on a common normalized gait cycle in which maximum hip flexion (MHF) and maximum hip extension (MHE) are placed at 0% and 60%, respectively, so that each profile is aligned to the event used to define its timing; the shaded regions and silhouettes indicate the stance (0-60%) and swing (60-100%) phases. The peak assistive force was set to 10% and 20% of body weight for hip flexion and hip extension, respectively, and the same action-specific profile was applied to both single-plane (SG) and multi-plane (MT) routing configurations.

We implemented a single-plane hip flexion assistance (FLX-SG) configuration using a monoarticular pathway from the front of the hip joint near the anterior superior iliac spine to the mid-anterior distal femur (10 cm superior to the knee joint line), thereby delivering hip-flexion torque only. This was compared with a multi-plane hip flexion assistance (FLX-MT) configuration that generated coordinated hip abduction and internal rotation through a biarticular cable pathway spanning from the same proximal anchor point to the anterolateral aspect of the proximal tibia near Gerdy’s tubercle, based on previously reported gait kinematics showing concurrent hip flexion, hip internal rotation, and knee flexion during the early- to mid-swing phase [22]. For hip extension assistance, a single-plane (EXT-SG) configuration used a monoarticular pathway spanning from the posterior pelvis near the gluteal surface to the mid-posterior distal femur, generating sagittal-plane hip extension torque only. The multi-plane hip extension (EXT-MT) configuration was designed to account for the coordinated hip extension and abduction moments generated during early to mid stance [22,23], which contribute to propulsion and lateral stability [24,25]. The proximal anchor point of EXT-MT was identical to that of EXT-SG, whereas the distal anchor point was positioned at the same location used in the FLX-MT configuration to create a lateral component in the cable line of action. The greater trochanter and fibular head served as anatomical landmarks for defining the proximal and distal attachment points, respectively. Bowden cables were used to transmit forces from the actuation unit to these defined points, which were integrated into the waist belt and thigh/knee wraps in a configuration designed to optimize the tradeoff between force capability and human-suit stiffness [26]. All configurations are presented in Fig 3 and Table 1.

**Table 1:** Summary of exosuit cable configurations.

Configuration	Condition	Proximal anchor	Distal anchor	Intended DOF	Expected moment direction
CON	Matched unpowered control	N/A	N/A	None (unassisted)	N/A
FLX-SG	Single-plane hip flexion	Anterior waist belt near anterior superior iliac spine (ASIS)	Mid-anterior distal femur (10 cm superior to knee joint line)	Hip flexion (sagittal only)	Hip flexion
FLX-MT	Multi-plane hip flexion	Anterior waist belt near ASIS (same as FLX-SG)	Anterolateral proximal tibia near Gerdy’s tubercle	Hip flexion, abduction, internal rotation (sagittal + frontal + transverse)	Hip flexion, abduction, internal rotation
EXT-SG	Single-plane hip extension	Posterior waist belt near gluteal surface of the pelvis	Mid-posterior distal femur (10 cm superior to knee joint line)	Hip extension (sagittal only)	Hip extension
EXT-MT	Multi-plane hip extension	Posterior waist belt near gluteal surface of the pelvis (same as EXT-SG)	Anterolateral proximal tibia near Gerdy’s tubercle (same as FLX-MT)	Hip extension, abduction, external rotation (sagittal + frontal + transverse)	Hip extension, abduction, external rotation

CON, matched unpowered control; FLX-SG, single-plane flexion; FLX-MT, multi-plane flexion; EXT-SG, single-plane extension; EXT-MT, multi-plane extension. ASIS, anterior superior iliac spine; DOF, degrees of freedom.

**Fig 3:**
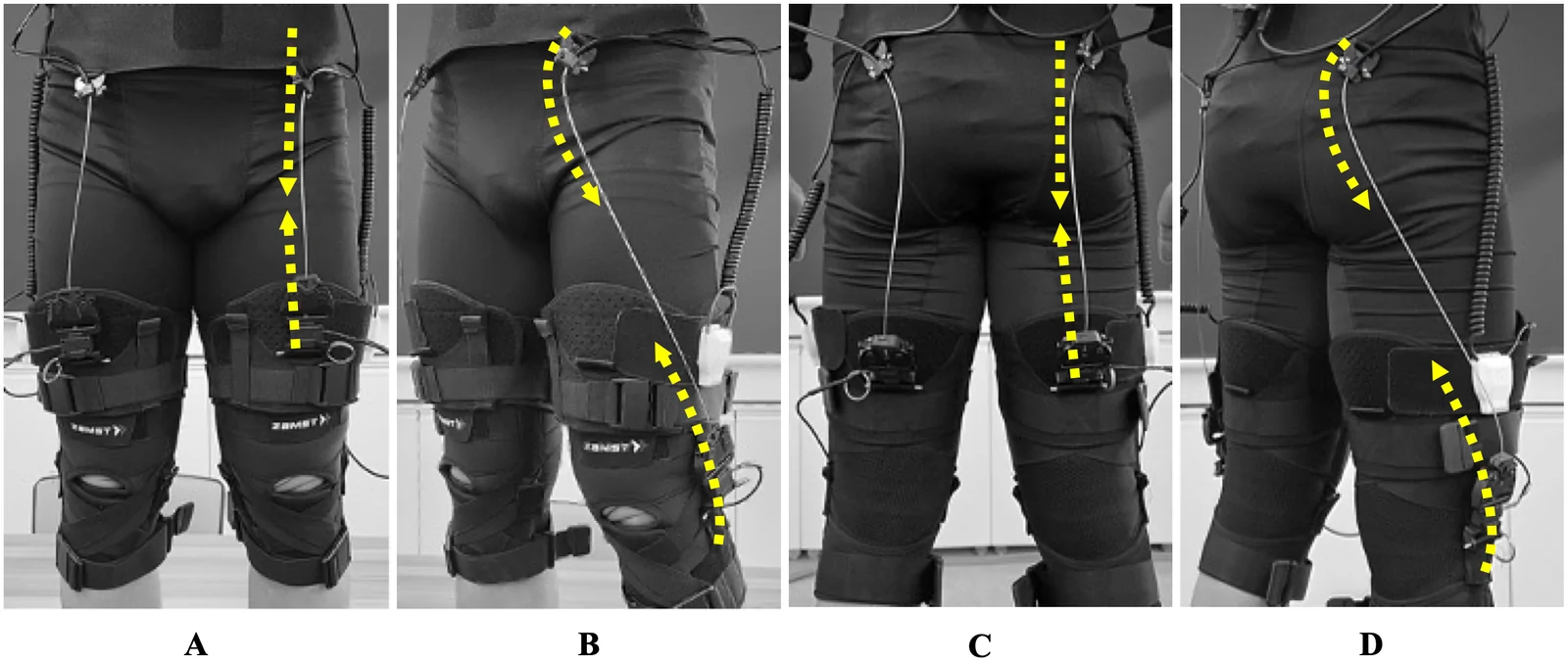
Four exosuit configurations for each assisted walking trial. (A) FLX-SG: the anchor points are on the front of the hip near the pelvis and on the mid-anterior part of the thigh; (B) FLX-MT: the anchor points are on the front of the hip near the pelvis and on the anterolateral aspect of the proximal tibia; (C) EXT-SG: the anchor points are on the back of the hip and on the mid-posterior part of the thigh; (D) EXT-MT: the anchor points are on the back of the hip and on the anterolateral aspect of the proximal tibia.

### Participants

The study involved 10 male adults with prior experience walking with the exosuit (age: 28.30 ± 1.06 years; height: 1.74 ± 0.04 m; body mass: 74.20 ± 7.44 kg). Participant recruitment and data collection were conducted from October to December 2025. Eligibility criteria required participants to be healthy individuals without walking difficulties, cardiac or respiratory conditions, or musculoskeletal injuries. Sex and sample size were constrained by the methodologically demanding and time-intensive nature of the study. The study design and protocol were approved by the Chung-Ang University Institutional Review Board (1041078–202107-HR-214-0). All participants provided written informed consent.

### Experimental setup and protocol

Each participant completed five experimental sessions, each consisting of 4 min of continuous level treadmill walking. The sessions included one matched unpowered control condition in which participants wore the same robot and garment as in the assisted conditions but received no active assistance (CON), and four powered conditions with distinct assistance configurations (FLX-SG, FLX-MT, EXT-SG, and EXT-MT).

Before the main experiment, each participant completed a preliminary session intended to confirm individual tolerance and comfort at the maximum assistive force for each configuration and to provide initial acclimation to the device, rather than to induce a lasting training effect. First, participants walked on a treadmill to familiarize themselves with the equipment at their self-selected preferred walking speed (1.0 ± 0.1 m/s). They were then instructed to walk under all experimental conditions with the maximum assistive force. The magnitude of this assistive force was determined on the basis of individual body weight (BW), specifically 10% BW for hip flexion and 20% BW for hip extension, following optimized profiles reported in prior studies. For hip flexion, Kim et al. [20] demonstrated that low assistance levels averaging approximately 10–15% BW were sufficient to reduce the metabolic cost of walking when timing was individually optimized, suggesting that meaningful biomechanical effects can be achieved without high force magnitudes. For hip extension, Ding et al. [21] employed a peak force of 30% BW in their human-in-the-loop optimization framework. The 20% BW used in the present study falls within a comparable range and was selected to balance sufficient assistance delivery with participant comfort across multiple experimental conditions.

The main experiment was conducted at least three days after the preliminary session. At the beginning of the main session, participants completed a 2-min familiarization trial to re-establish comfortable exosuit and treadmill walking on the test day. All experimental trials were conducted at a constant speed of 1.0 m/s on an instrumented treadmill, with the maximum assistive force applied as previously defined. The CON condition was always performed first to establish a matched unpowered baseline uncontaminated by prior active assistance. This ordering was intentional because preceding powered conditions may induce locomotor adaptation that alters baseline gait kinematics, thereby compromising the validity of the unpowered reference [27]. Because CON was not counterbalanced, its comparison with the assisted conditions may be partly confounded by progressive familiarization and cumulative walking exposure. However, ordering or fatigue alone cannot account for the transverse-plane MT effects, because these effects depended on the specific routing direction rather than appearing uniformly across all assisted conditions. The remaining experimental conditions were then performed in randomized order, with the previously defined maximum assistive force, to minimize carryover effects. Randomization followed a hierarchical approach: the assistance phase (swing versus stance) was determined first, followed by the assistance plane (SG versus MT) within each phase. The order was independently randomized for each participant using a random-number generator, with no constraints imposed on the sequence. Between consecutive trials, a minimum rest period of 5 min was provided to mitigate peripheral fatigue of the lower limbs. Participants were instructed to walk in the manner they found most comfortable, and they received no additional external feedback regarding lower-limb kinematics or kinetics. All walking trials were recorded using a markerless motion capture setup, which is described in detail in the following section. The same setup was maintained across all experimental conditions to ensure consistency of the recorded motion data, as shown in Fig 4A and B. A video overview of the experimental protocol and representative treadmill walking under the four assisted configurations is provided in S1 File. The experimental protocol described in this section is also published on protocols.io, https://dx.doi.org/10.17504/protocols.io.e6nvwxk87gmk/v1.

**Fig 4:**
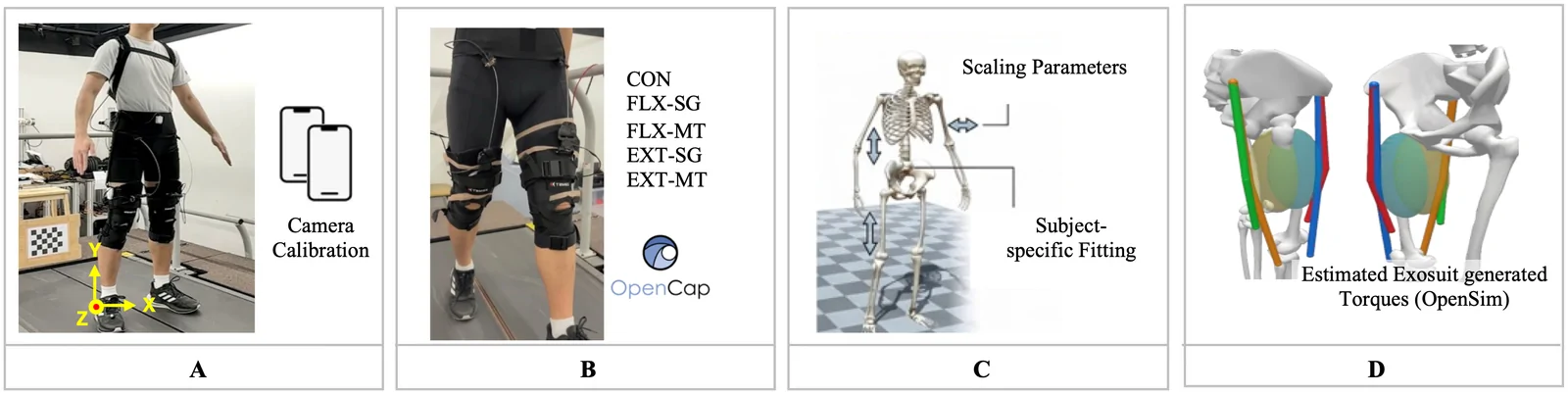
Brief overview of the experimental setup and protocol. (A) Static trial with a participant standing in a neutral A-pose after camera calibration. (B) Treadmill walking trial performed under the five conditions (CON, FLX-SG, FLX-MT, EXT-SG, EXT-MT) using the markerless OpenCap platform. (C) Subject-specific musculoskeletal model scaling. (D) An analysis on estimated exosuit-generated coordinate torques using OpenSim. Photographs in Panels A and B published under a CC BY 4.0 license, with written permission from the individual depicted.

### Data collection and processing

Traditional marker-based motion capture was precluded because the exosuit’s anchor points and actuation forces caused occlusion and movement of key landmarks, such as the femoral epicondyles. The compromised tracking integrity of these markers could severely degrade the reliability of kinematic measurements [28,29]. To overcome this limitation, we used the markerless OpenCap platform [30] with OpenPose [31] as the underlying pose estimation model for three-dimensional musculoskeletal modeling and kinematic analyses for all walking trials. OpenCap is a smartphone-based open-source system that computes joint kinematics and kinetics of human movement from two or more cameras. Given the marker occlusion and displacement caused by the exosuit, the use of OpenCap in the present study was considered methodologically appropriate. Previous validation work [32] reported that, during overground walking, the OpenCap showed hip-joint RMSE values of 5.4° (SD 2.1°) in the sagittal plane, 3.7° (SD 1.0°) in the frontal plane, and 6.6° (SD 2.7°) in the transverse plane, relative to marker-based motion capture in healthy populations. This accuracy of OpenCap is comparable to that of other accessible motion-analysis approaches. For example, a commercial eight-camera markerless system reported RMSE values below 5.5° for most segment angles [33] and IMU-based methods for knee kinematics have reported RMSEs ranging from 3.9° to 7.8° depending on the calibration approach [34]. These comparisons further support the use of OpenCap as a practical tool for gait analysis in the present study. Accordingly, because the exosuit hardware could occlude and displace reflective markers, leading to marker gaps and noisy data, the OpenCap system provided a practical and methodologically appropriate alternative for measuring joint kinematics in this study.

OpenCap was used to record video from two smartphones (iPhone 13 Pro and iPhone 15, Apple Inc., Cupertino, CA, USA). The smartphones were positioned 1.7 m above the base of the instrumented treadmill and 2.5 m from the participant and were arranged at 45° intervals around the participant. This two-camera count and placement followed the configuration recommended for OpenCap lower-limb gait capture [30]. For camera calibration, a checkerboard pattern (4 × 5 grid with 35-mm squares) was used. A single video frame capturing the checkerboard within the shared field of view was used to estimate the spatial position and orientation of each camera relative to the global coordinate system. A static trial was subsequently recorded with the participant standing in a neutral A-pose. Virtual marker trajectories derived from this static trial were used as inputs to OpenSim’s Scale Tool to scale a generic musculoskeletal model [35,36] to each participant’s anthropometry. The resulting subject-specific model had 33 degrees of freedom, as shown in Fig 4C. In this generic gait model, the knee was represented as a single sagittal-plane (flexion-extension) degree of freedom, so three-dimensional knee kinematics could not be derived from the markerless data.

Gait events were identified from foot-marker trajectories projected onto the progression axis, defined from the pelvis (ASIS/PSIS) landmarks. Heel strike (HS) was defined as a local maximum of the anteroposterior calcaneus-marker position projected onto this axis, and toe-off (TO) as a local maximum of the inverted toe projection (the anteroposterior toe-marker position multiplied by −1, so that toe-off appears as a local maximum). Peak detection used adaptive prominence thresholds of 0.3, 0.25, and 0.2, and the detected events were required to follow the alternating sequence of right HS, left TO, left HS, right TO, or the mirrored left-right sequence. The ten consecutive strides used for averaging were drawn from the final minute of each 4-min trial to ensure steady-state walking. Across these strides, the stride-to-stride coefficient of variation of the hip range of motion averaged approximately 5% in the sagittal plane, 7% in the frontal plane, and 16% in the transverse plane across conditions. The higher relative variability in the transverse plane reflects its comparatively small range of motion. These strides were averaged to obtain mean joint-angle trajectories, and the filtered joint-angle data were then time-normalized to the gait cycle. Toe-off was detected individually for every stride from each participant’s own marker trajectories, so stance and swing were segmented on a participant- and stride-specific basis. Mean toe-off occurred at 67–68% of the gait cycle across conditions (CON, 67%; FLX-SG, 68%; FLX-MT, 68%; EXT-SG, 67%; EXT-MT, 68%), with a between-participant standard deviation of approximately 1–2% of the gait cycle in each condition. The single mean toe-off shown for each condition serves only as a reference marker on the group-averaged waveforms. Mean trajectories and the associated standard deviations (SDs) were computed and plotted for each condition.

To provide a mechanistic interpretation of the observed kinematic changes, estimated exosuit-generated coordinate torques were additionally obtained using OpenSim. In addition to OpenCap-based model scaling, thigh circumference was measured for each participant at the cable-wrapping region and used to parameterize a subject-specific wrap cylinder in OpenSim, as shown in Fig 4D. For each assistance configuration, the exosuit cable was modeled as a path actuator connecting the experimentally defined proximal and distal anchor locations while wrapping around the thigh cylinder to reproduce the intended routing geometry. Subject-specific musculoskeletal models scaled from OpenCap data were combined with the experimentally measured assistive force recorded by the load cells. Coordinate-specific moment arms about the hip sagittal, frontal, and transverse coordinates were then computed across the observed gait kinematics. Estimated exosuit-generated coordinate torques were calculated as the product of cable tension and instantaneous moment arm at each time point. These waveforms were used as descriptive biomechanical indicators of the direction and timing of exosuit assistance over the gait cycle.

### Data analysis and statistics

First, statistical parametric mapping (SPM) was applied to examine significant differences in joint kinematics among the four experimental conditions across the normalized gait cycle (0%−100%), as well as between the corresponding assistance planes (SG versus MT) [37,38]. Unlike conventional scalar-based analyses that rely on the ad-hoc extraction of discrete variables (e.g., peaks or means), SPM enables an unbiased, continuous statistical evaluation over the entire one-dimensional waveform. A repeated-measures SPM{F} analysis (one-way repeated measures ANOVA) was performed using the spm1d package (version 0.4.53, spm1d.org) in Python to test the null hypothesis $H_{\text{0}}:\;\mu_{\text{1}}(t)=\mu_{\text{2}}(t)=\cdots =\mu_{\text{5}}(t$at each time point t of the gait cycle. SPM employs Random Field Theory (RFT) to control the family-wise error rate by accounting for the temporal smoothness of the data, thereby identifying supra-threshold clusters where the F-statistic exceeds the critical threshold $F_{\alpha =\text{0}.\text{0}\text{5}}^{*}$. When a significant main effect was detected, post-hoc pairwise SPM{t} tests were subsequently conducted between conditions to locate the specific temporal regions of difference, with Holm-Bonferroni correction applied within each plane across the six pairwise contrasts examined (each assisted condition versus CON, plus FLX-SG versus FLX-MT and EXT-SG versus EXT-MT). For each participant and condition, ten consecutive strides were time-normalized and averaged to obtain a single representative waveform, which was then entered into the SPM analysis. Paired effect sizes (Cohen’s d_z_) were computed for the significant SPM post-hoc contrasts. To benchmark the observed effects against within-session variability, we additionally computed the stride-to-stride standard deviation of each hip angle within each condition, averaged across the gait cycle and participants. All data processing and statistical analyses were performed using custom scripts written in Python (version 3.10.19).

Additionally, to evaluate the similarity of joint kinematics with and without robotic assistance, we used Dynamic Time Warping (DTW), following the approach of Lambranzi et al. [17] for exosuit analysis. Even for waveforms normalized to a common gait-cycle length, DTW provides an alignment-robust similarity measure that is less sensitive to residual phase differences than point-wise metrics. The DTW algorithm was developed to address pattern similarity for time-series data, and has utilized in analyzing gait pattern similarity [39,40]. For each participant and condition, multiple strides were time-normalized and averaged to obtain a representative joint-angle waveform. DTW was then computed at the participant level by comparing each assisted-condition waveform with the corresponding matched unpowered control (CON) waveform from the same participant. DTW distances were computed for the three hip-plane joint-angle variables: hip flexion (sagittal), hip adduction (frontal), and hip rotation (transverse). The DTW distance between sequences $X=\left(x_{\text{1}},x_{\text{2}},\ldots ,x_{n}\right)$ and $Y=\left(y_{\text{1}},y_{\text{2}},\ldots ,y_{m}\right)$ was defined recursively as in (1).


$$\begin{array}{c} DTW(i,j)=\left|x_{i}-y_{j}\right|+\text{min}\left\{ \begin{array}{c} DTW(i-\text{1},j),DTW(i,j-\text{1}),\\ DTW(i-\text{1},j-\text{1}) \end{array}\right. \end{array}$$ (1)

Here, $\left|x_{i}-y_{j}\right|$ represents the absolute point-wise difference between the i-th and j-th elements of X and Y, respectively. The final DTW distance was given by d=DTW(n,m), with lower values indicating greater similarity between trajectories. DTW distances were treated as scalar similarity metrics and entered into subsequent statistical analyses to compare the relative similarity of assisted conditions to unassisted walking. DTW distances, computed relative to the participant-specific control condition, were analyzed within a within-subject framework.

Finally, to complement the time-series-based similarity analysis, a change-based peak kinematic analysis was performed to quantify discrete alterations in joint kinematics induced by exosuit assistance. Peak joint angles were extracted from the time-normalized mean gait cycle for each participant and condition. To isolate the effect of robotic assistance while accounting for inter-individual baseline differences, peak-angle changes were computed relative to unassisted walking (Δ = Assisted – CON) on a participant-specific basis. The resulting change scores were treated as paired observations. The knee and ankle were examined only exploratorily and were not included in the reported DTW or peak analyses, because the single-degree-of-freedom knee representation and the lower reliability of markerless distal-joint estimates precluded their confident interpretation [41]. For both DTW and change-based peak analyses, paired nonparametric comparisons were conducted using Wilcoxon signed-rank tests, with Holm-Bonferroni correction applied across all comparisons within each analysis. The DTW analysis comprised 6 comparisons, the two SG-versus-MT contrasts across the three hip-plane variables. The peak analysis comprised 12 comparisons, two contrasts and two peak directions across the three hip-plane variables. All statistical tests were two-sided with a significance threshold of α = 0.05. For the significant DTW and peak contrasts, effect sizes are reported as the matched-pairs rank-biserial correlation r, the effect-size measure appropriate for the Wilcoxon signed-rank test. Change-based peak values are reported as median and interquartile range unless otherwise stated.

The processed stride-averaged joint-angle waveform datasets and statistical analysis outputs from the DTW and the peak analyses used in this study are provided in the S2-S4 Data. The custom analysis code (OpenSim processing and the SPM, DTW, and peak-analysis scripts) is publicly available at https://doi.org/10.6084/m9.figshare.32918876.

## Results

### Continuous joint angle patterns across the gait cycle

SPM analysis revealed significant main effects of condition for multiple hip joint angles across the gait cycle. Accordingly, post hoc SPM{t} analyses were conducted to identify condition-specific differences, as shown in Fig 5A-C. For the hip sagittal-plane angle, post hoc analyses showed that, relative to CON, FLX-MT exhibited a more flexed hip posture during part of the gait cycle (3.5°, p = 0.0095, d_z_ = 1.52), whereas EXT-MT showed a more extended hip posture than CON (2.5°, p = 0.0007, d_z_ = 1.89). Within the flexion-assisted conditions, FLX-MT induced greater hip flexion than FLX-SG, indicating larger sagittal-plane deviations associated with the MT configuration (2.7–2.8°, p < 0.05). For the hip frontal-plane angle, post hoc analyses revealed no significant differences between CON and any assisted condition. A significant difference was observed only within the flexion-assisted conditions, in which FLX-MT demonstrated a more abducted hip position than FLX-SG during a portion of the gait cycle (1.2°, p = 0.0204). No significant differences were detected between EXT-SG and EXT-MT. For the hip transverse-plane angle, post hoc SPM{t} analyses showed that FLX-MT produced greater internal hip rotation relative to CON (3.6°, p = 0.0002, d_z_ = 1.55), whereas EXT-MT resulted in greater external rotation than CON (1.8°, p = 0.0095, d_z_ = 1.87). A smaller but significant difference was also observed between CON and FLX-SG, characterized by slightly greater external rotation (1.1°, p = 0.0463, d_z_ = 1.31). Comparisons within assistance types further indicated that FLX-MT elicited larger internal rotation than FLX-SG, whereas EXT-MT demonstrated greater external rotation than EXT-SG, suggesting that MT routing amplified transverse-plane kinematic deviations without altering the overall direction associated with each assistance mode. For reference, within-condition stride-to-stride variability of the hip angles averaged 1.1° in the transverse plane, 1.3–1.6° in the sagittal plane, and 0.8–0.9° in the frontal plane across conditions. Within-session repeatability metrics, including intraclass correlation coefficients and minimal detectable change estimates at the 95% confidence level (MDC₉₅), are reported in S5 File.

**Fig 5:**
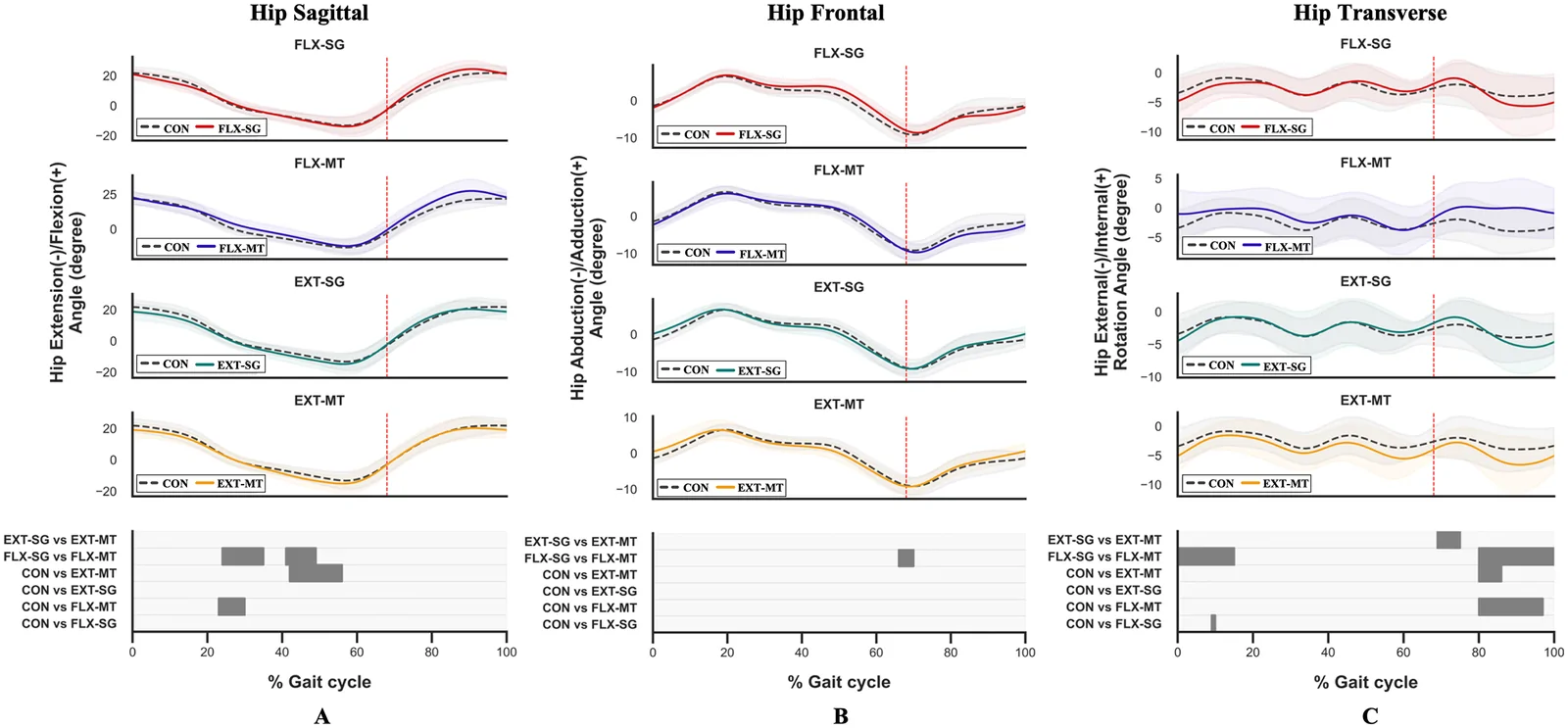
Three-dimensional hip joint kinematics across all experimental conditions. Mean hip joint angles in (A) sagittal, (B) frontal, and (C) transverse planes are shown with standard deviation for the different configurations compared to matched unpowered CON. The one-way repeated-measures SPM{f} analysis was performed. However, only the post-hoc pairwise SPM{t} results are shown. Line colours denote condition: CON (dark grey, dashed), FLX-SG (red), FLX-MT (blue), EXT-SG (teal), and EXT-MT (orange).

### Estimated exosuit-generated torques

OpenSim-based estimates of exosuit-generated torques at the hip showed that all assistance conditions produced dominant sagittal-plane torques in the intended direction, as shown in Fig 6A-C. During flexion assistance, both FLX-SG and FLX-MT generated late-swing hip-flexion torques, reaching 6.38 ± 0.55 Nm and 5.72 ± 0.80 Nm, respectively. During extension assistance, both EXT-SG and EXT-MT generated early- to mid-stance hip-extension torques, reaching −11.42 ± 1.73 Nm and −10.02 ± 0.18 Nm, respectively.

**Fig 6:**
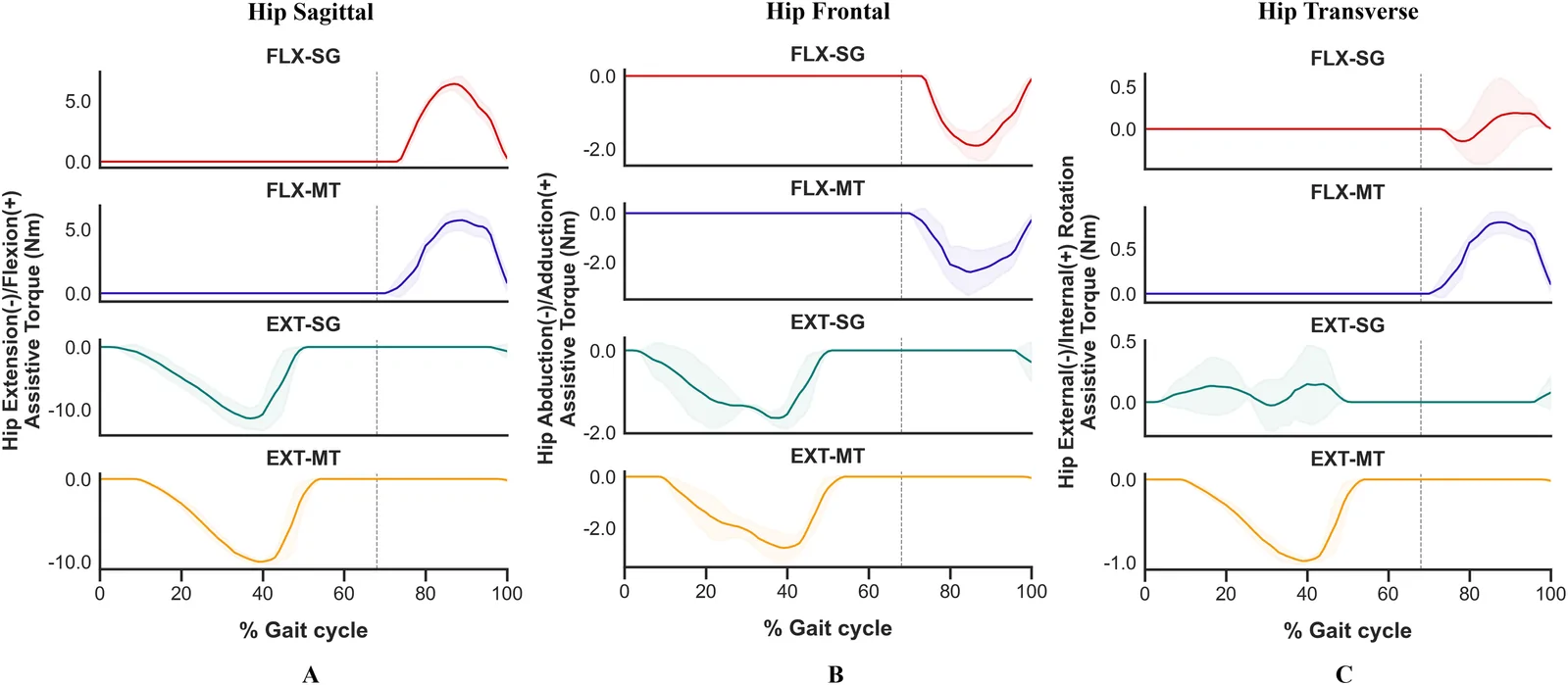
Three-dimensional profiles of estimated exosuit-generated hip assistive torque across four assisted conditions. Mean estimated exosuit-generated torques about the hip joint in the (A) sagittal, (B) frontal, and (C) transverse planes are shown with standard deviation across the gait cycle for the four assistance configurations. Positive values represent flexion, adduction, and internal rotation torques, whereas negative values represent extension, abduction, and external rotation torques. The vertical dashed line denotes toe-off.

Torque components in the frontal and transverse planes differed across routing configurations. In the frontal plane, both flexion-assisted conditions generated late-swing hip-abduction torques, with peak values of −1.91 ± 0.38 Nm in FLX-SG and −2.41 ± 0.91 Nm in FLX-MT. During extension assistance, both EXT-SG and EXT-MT also generated early- to mid-stance hip-abduction torques, with peak values of −1.65 ± 0.23 Nm and −2.78 ± 0.57 Nm, respectively. In the transverse plane, FLX-SG showed a late-swing internal-rotation torque of 0.19 ± 0.30 Nm, whereas FLX-MT showed a substantially larger internal-rotation torque of 0.79 ± 0.12 Nm. During extension assistance, EXT-SG showed only a trivial transverse-plane component, whereas EXT-MT generated a clear early- to mid-stance external-rotation torque of −0.98 ± 0.04 Nm.

### Kinematics similarity to unassisted walking

Across the three hip planes, the hip frontal plane during extension assistance was the only SG-versus-MT comparison to reach statistical significance after Holm-Bonferroni correction (adjusted p = 0.035, r = 0.93; Fig 7B), where MT produced a greater departure from the participant-specific unpowered reference than SG. All other comparisons were non-significant (all other adjusted p ≥ 0.42; Fig 7A-C). In the flexion-assisted conditions, DTW distances for MT were generally larger than those for SG in the hip sagittal plane, suggesting greater departures from the unpowered reference. However, these differences did not remain statistically significant following correction for multiple comparisons. In the extension-assisted conditions, SG-versus-MT DTW differences were small in the sagittal and transverse planes but were larger, and statistically significant, in the frontal plane. Overall, the DTW analysis indicates that assistance configuration (SG versus MT) yielded a statistically distinguishable difference in time-series similarity to unassisted walking only in the hip frontal plane during extension, with modest and non-significant trends elsewhere.

**Fig 7:**
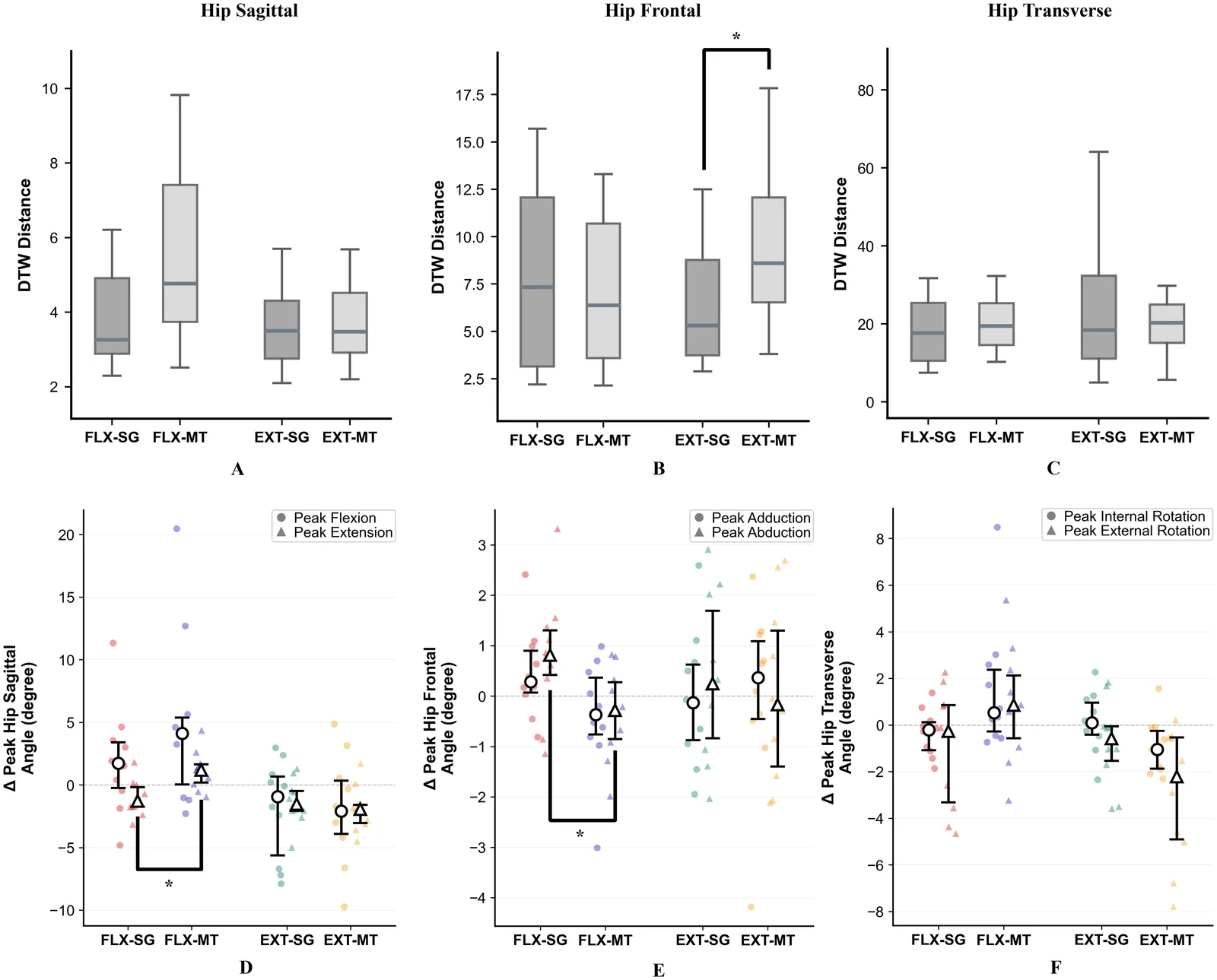
Three-dimensional hip joint kinematic similarity and peak-angle changes relative to matched unpowered CON. The figure is organized in two rows and three columns, with columns corresponding to the (A and D) sagittal, (B and E) frontal, and (C and F) transverse planes. The top row A-C shows DTW distances between each assisted-condition waveform (FLX-SG, FLX-MT, EXT-SG, EXT-MT) and the participant-specific CON waveform, where lower values indicate greater similarity to unassisted walking. The bottom row D-F presents changes in peak hip joint angles relative to CON for each assistance configuration. Within each plane, circles and triangles denote direction-specific peak variables: flexion and extension (sagittal), adduction and abduction (frontal), and internal and external rotation (transverse). Colored symbols represent individual participants, while open symbols and error bars indicate the mean and standard deviation. Y-axis ranges differ across planes to accommodate the different DTW-distance magnitudes, so absolute comparisons across planes should be made with caution. Brackets marked with an asterisk denote statistically significant SG-versus-MT differences after Holm-Bonferroni correction (* adjusted p < 0.05): the DTW distance in the frontal plane under extension assistance (B), and the peak hip extension (D) and peak hip abduction (E) changes under flexion assistance.

### Discrete changes in peak joint angles

Two discrete peak measures differed significantly between SG and MT after Holm-Bonferroni correction, both during flexion assistance. Peak hip extension was greater under SG than under MT, because relative to CON the peak extension angle increased under SG but decreased under MT, a difference of 1.6° (median; mean 2.2°) between configurations (adjusted p = 0.047, r = 0.96; Fig 7D). Peak hip abduction was greater under MT than under SG, because relative to CON peak abduction increased under MT but not under SG, a difference of 0.8° (median; mean 1.1°) between configurations (adjusted p = 0.047, r = 0.96; Fig 7E). For the remaining contrasts, MT generally produced numerically larger peak changes than SG, although none reached significance. Expressed as SG-versus-MT differences, these were 3.9° (median; mean 3.1°) for peak hip flexion during flexion assistance, 0.6° (median; mean 0.9°) for peak hip adduction during flexion assistance, and 1.3° (median; mean 2.2°) for peak hip external rotation during extension assistance. None of the remaining contrasts reached statistical significance after adjustment for multiple comparisons (all other adjusted p ≥ 0.27).

## Discussion

This study investigated how biarticular MT routing (multi-plane) versus monoarticular SG routing (single-plane) in a hip-assisting exosuit shapes lower-limb gait kinematics during treadmill walking. The results suggest that the MT configuration produced localized, phase-dependent 3-D kinematic deviations, with the clearest and most consistent effects emerging in hip transverse-plane rotation. Between SG and MT, overall trajectory similarity and discrete peak changes remained largely comparable after multiple-comparison correction, with significant differences confined to the frontal-plane DTW distance during extension and to two discrete peaks during flexion. An analysis of estimated exosuit-generated hip torques provided a mechanical interpretation of these kinematic changes by characterizing the timing, direction, and plane-specific composition of the assistance.

The sagittal-plane SPM analysis showed that FLX-MT increased hip flexion during early to mid stance, whereas EXT-MT increased hip extension during mid stance relative to CON. The estimated exosuit-generated torque analysis indicated that all four configurations were dominated by sagittal-plane assistance in the intended direction. Although late-swing differences between FLX-SG or FLX-MT and CON did not reach statistical significance in the SPM analysis, both flexion-assisted conditions visually showed greater hip flexion than CON during swing. Biomechanically, the biarticular MT routing likely changed how the assistive force interacted with limb posture across the gait cycle, rather than simply increasing sagittal assistance magnitude. Because the oblique MT pathway alters the hip moment arm, the same cable tension produced slightly lower estimated peak sagittal-plane torques under MT than SG (FLX-MT 5.72 vs. FLX-SG 6.38 Nm; EXT-MT −10.02 vs. EXT-SG −11.42 Nm), so a matched force profile did not equate to matched sagittal-plane assistance across routing configurations. MT routing produced greater sagittal-plane hip excursion than SG despite this lower estimated sagittal torque, so the additional excursion cannot be attributed to a larger sagittal assistance magnitude. Absent direct kinetic measurement, we treat this torque-kinematics dissociation as provisional. This interpretation is consistent with prior work showing that robotic assistance can induce localized gait adaptations [27] and that hip-flexion assistance can cause participants to walk with consistently greater hip flexion across the gait cycle [42].

In the frontal plane, SPM revealed only limited kinematic differences between the assisted conditions and CON. A small but significant difference was observed only between FLX-SG and FLX-MT during pre- to early swing, with FLX-MT showing slightly greater hip abduction. Consistent with this pattern, the estimated exosuit-generated torque analysis showed that both flexion- and extension-assisted conditions included hip-abduction torque components and that these components were larger in the MT configurations. This overall pattern provides only limited support for the first hypothesis in the frontal plane, suggesting that multi-plane routing introduced a measurable frontal-plane assistance component, but that its kinematic expression remained modest during steady treadmill walking. The lack of significant frontal-plane kinematic changes during stance suggests that, although EXT-MT introduced a larger abduction-torque component, this assistance was still insufficient to meaningfully alter pelvic stabilization. During stance, frontal-plane hip control depends largely on the internal abductor moment, which counteracts the external adduction moment generated by body weight [24]. One possible explanation is that the frontal-plane assistance component remained small relative to the native abductor demand during stance. This interpretation is supported by Park et al. [9], who showed that frontal-plane hip assistance can influence balance-related variables and metabolic cost when hip-abduction torque is explicitly targeted and timed to the biological moment profile. Their findings suggest that meaningful frontal-plane effects require more deliberate assistance timing and direction than the indirect abduction component generated in the present study. For a mean body mass of 74.2 kg, the ~ 2.78 Nm peak indirect abduction torque generated by EXT-MT is roughly an order of magnitude smaller than the peak internal hip-abductor moment required to stabilize the pelvis during stance in healthy adults [24,43]. Consequently, the indirect frontal-plane components introduced by multi-plane routing were likely too small to produce a clear stance-phase kinematic effect against the substantial native abductor demand.

The transverse-plane SPM results showed the clearest MT-specific kinematic effects. FLX-MT increased internal rotation during late swing, whereas EXT-MT increased external rotation relative to CON and SG. This pattern was consistent with the estimated exosuit-generated torque analysis, which we report as descriptive context for the direction and timing of assistance rather than as independent confirmation, because both were derived from the same OpenCap kinematics. FLX-MT generated a larger late-swing internal-rotation torque than FLX-SG, whereas EXT-MT generated a clear early- to mid-stance external-rotation torque, and EXT-SG showed only a minimal transverse-plane component. This supports the first hypothesis and suggests that multi-plane routing can alter the rotational assistance delivered about the hip. The oblique cable pathway appears to alter the transverse-plane moment arm as the thigh advances. Because the MT cable connects pelvic and anterolateral tibial landmarks, small changes in segment orientation may generate rotational moments around the hip. This may create a direction-specific internal- or external-rotation bias, depending on assistance mode. This view is in line with Zhang et al. [18], who showed that modifying the assistive force line can change multidimensional ankle-joint kinematics. Our findings extend this concept to the hip and suggest that force-line routing designed to generate rotational moments can influence transverse-plane kinematics. From a practical perspective, MT routing may offer a useful strategy for influencing transverse-plane kinematics, particularly in clinical populations exhibiting rotational gait deviations. For example, children with cerebral palsy commonly exhibit excessive hip internal rotation during stance compared with typically developing children [44]. The 1.8° external-rotation increase produced by EXT-MT represents a corrective direction relative to this deviation, suggesting that stance-phase multi-plane routing may offer a meaningful assistive component for reducing pathological internal rotation during weight-bearing. However, because reported internal-rotation deviations in this population can range from 5° to 8°, the magnitude observed in the present study likely represents only a partial correction, and its clinical significance should be evaluated in target populations in future work. Targeted exosuit assistance has previously been shown to modify specific pathological gait patterns rather than uniformly changing the entire gait cycle [15].

Kinematic differences from CON also appeared in phases where the estimated assistive torque was near zero (e.g., FLX-MT during stance and EXT-MT during swing). These differences may reflect passive mechanical interaction between the garment and limb, biarticular coupling transmitted through the knee-crossing MT cable, or within-cycle carryover in which assistance delivered in one phase altered the limb state entering the next. CON was a matched unpowered condition in which participants wore the same robot and garment without active assistance. Therefore, comparisons with CON controlled for the effects of wearing the device and passive garment-limb interaction under the unpowered condition. Nevertheless, the temporal relationship between the estimated torque profile and the observed deviations provides an additional line of evidence regarding the contribution of active assistance. A post hoc phase-stratified analysis of deviations from CON revealed contrasting directional patterns between the two multi-plane conditions, as shown in S6 File. Specifically, deviations were larger during torque-active phases under FLX-MT, while under EXT-MT, they were larger during near-zero-torque phases. This aligns with the within-cycle carryover interpretation. Passive garment-limb coupling alone is unlikely to explain these distinct temporal patterns because its effects were represented in the matched unpowered CON condition. The post hoc results therefore support active assistance as the primary contributor to the observed kinematic differences, while the near-zero-torque deviations under EXT-MT remain consistent with within-cycle carryover. Direct measurements of joint kinetics and garment-limb interface forces would be required to quantify the mechanical pathways underlying these responses.

The main finding of the DTW analysis was that SG and MT differed significantly in only one of the six comparisons, the hip frontal plane during extension assistance, while the remaining comparisons were not significant. This largely supports our second hypothesis that MT assistance would preserve overall gait-trajectory similarity to unassisted walking to an extent broadly comparable to that of SG assistance, with the frontal plane during extension as a partial exception. This frontal-plane exception reflects how much more EXT-MT departed from the participant-specific unpowered reference than EXT-SG, rather than a significant frontal-plane deviation from unassisted walking itself. It is consistent with the SPM analysis, in which no assisted condition differed significantly from CON in the frontal plane, and it does not change the characterization of the frontal-plane effects as indirect and sub-significant. Although localized kinematic differences were observed, these did not translate into large whole-waveform dissimilarities. The estimated exosuit-generated torque analysis offers a plausible explanation for this pattern. The additional frontal- and transverse-plane torque components introduced by MT routing were smaller than the dominant sagittal-plane component and were therefore more likely to produce localized deviations than large whole-cycle differences. With the single exception of the hip frontal plane during extension, the DTW comparisons showed no significant SG-versus-MT differences, indicating that multi-plane routing did not broadly reduce overall movement similarity to unassisted walking relative to SG routing. One possible explanation is that participants accommodated the MT-induced, plane-specific perturbations during walking while preserving the overall temporal structure of the joint-angle trajectories. This explanation aligns with Gordon and Ferris [27], who showed that powered robotic assistance initially disrupted gait kinematics, but that practice allowed participants to restore kinematic patterns toward baseline through locomotor adaptation. Another previous study supports this view by showing that multi-joint soft-exosuit assistance introduced only minimal changes in gait kinematics while still reducing biological joint moments [45]. In this context, the present DTW findings suggest that MT assistance preserved overall gait trajectories without overt disruption of joint-level kinematic organization.

The peak analysis showed two significant SG-versus-MT differences during flexion assistance, in peak hip extension (greater under SG) and peak hip abduction (greater under MT), whereas the remaining discrete comparisons showed only non-significant numerical trends. This confinement of significant effects to specific peaks suggests that MT assistance had phase-specific effects rather than producing a uniform shift in peak magnitudes. The significant peak effects appeared during flexion assistance, whereas the significant waveform-level DTW difference appeared in the frontal plane during extension, indicating that the two analyses were sensitive to different localized aspects of the routing-induced changes. This interpretation is consistent with Lambranzi et al. [17], who reported that exosuit anchor-point placement affected multi-joint kinematic trajectories, whereas the corresponding discrete maximum and minimum values were less sensitive to those differences. One possible explanation is that individual responses to the actuator increased between-subject variability, which may affect discrete peak values more strongly than time-localized waveform features. In this context, the peak-analysis results reinforce the view that MT routing modified specific portions of the gait cycle without producing a large global shift in representative peak angles.

For future exosuit routing design, greater attention may need to be given to the spatial geometry of the force line rather than to torque magnitude alone. The present results suggest that multi-plane routing did not markedly disrupt overall gait-trajectory similarity, but it did introduce phase-dependent frontal- and transverse-plane effects at the hip. This implies that cable pathways can be shaped to modify three-dimensional assistance profiles across the gait cycle. Rather than simply increasing assistance strength, future designs may benefit from strategically adjusting proximal and distal anchor placement and cable obliquity to modulate rotational or frontal-plane components while preserving sagittal-plane support. Such an approach may be particularly useful in clinical populations with abnormal frontal- or transverse-plane gait mechanics.

Several limitations should be considered when interpreting the present findings. First, the main outcomes were kinematic, and joint kinetics and muscle demand were not directly evaluated because of experimental constraints. Consequently, the present data cannot establish whether the routing-induced kinematic changes, or their absence, reflect a true motor adaptation rather than measurement variability. Force-plate inverse dynamics or electromyography would be required to make that distinction. Second, the estimated exosuit-generated hip torques were descriptive model-based quantities derived from measured cable tension and modeled moment arms and should not be interpreted as direct measures of biological loading. Third, because the torque estimates depended on OpenCap-based kinematics and subject-specific cable-path modeling, errors in kinematic reconstruction, scaling, or routing geometry may have influenced the estimated torque profiles. In addition, the MT cable is biarticular and crosses the knee as well as the hip, so the hip kinematic differences attributed to routing geometry may partly reflect knee-mediated effects propagated along the kinematic chain. As noted, the single-DOF knee model precluded the three-dimensional knee kinematics and knee-level torques that would be needed to separate the direct hip action from indirect knee-mediated contributions.

The accuracy of OpenCap under exosuit-wearing conditions was not independently validated, and the transverse-plane effects reported here (1.1°-3.6°) are smaller than OpenCap’s published single-trial transverse-plane accuracy RMSE of 6.6° against marker-based reference [32]. That RMSE reflects between-system accuracy, which does not reflect the within-subject repeatability that matters for these repeated-measures contrasts. Interpreted against the within-session stride-to-stride variability reported above (approximately 1.1° in the transverse plane), which conservatively bounds measurement noise together with genuine biological fluctuation, the principal transverse effect (FLX-MT, 3.6°) and the sagittal effects exceed this variability. However, because the smallest transverse effect (FLX-SG, 1.1°) is comparable in magnitude to this variability, it warrants cautious interpretation. To formally quantify measurement precision, we computed within-session ICC(2,1) using a split-half approach (odd versus even strides) across all conditions and participants. The resulting ICC values ranged from 0.944 (transverse) to 0.998 (sagittal), yielding a MDC₉₅ of 0.19° (sagittal), 0.22° (frontal), and 0.70° (transverse). All seven statistically significant effects reported in this study exceeded their respective MDC₉₅ thresholds by factors ranging from 1.6 to 18.4 (as reported in S5 File). Notably, even the smallest of these effects exceeded its corresponding MDC₉₅, indicating that all reported effects surpassed the estimated within-session measurement error threshold, even when their magnitudes approached the stride-to-stride SD. Two-camera markerless capture may further limit transverse-plane resolution, the plane most challenging for markerless systems, reinforcing this cautious reading. For this reason, the transverse-plane results should be read as phase-localized statistical effects whose magnitude is comparable to the measurement uncertainty, and a dedicated repeatability assessment under exosuit-wearing conditions is still needed.

The matched unpowered control condition was always recorded first to protect the unpowered reference from prior-assistance carryover. As a result, the powered conditions were performed after more treadmill familiarization and with greater accumulated walking time than the control, so order, familiarization, or fatigue effects cannot be fully separated from routing effects in the matched unpowered control comparisons. In addition, the assisted conditions were assigned by unconstrained randomization rather than a formal counterbalanced sequence, which, given the small sample, does not guarantee balanced ordering across participants. No a priori power analysis was conducted. A sensitivity analysis indicated that this paired design (n = 10, α = 0.05, two-sided) had approximately 80% power only for within-subject effects of Cohen’s d_z_ ≈ 1.0 or larger. Under this constraint, the comparisons that did not reach significance may be underpowered and should be interpreted with caution. These include the frontal-plane MT-versus-CON contrasts and the remaining DTW and peak comparisons. In addition, all participants were male and experienced with exosuit walking, so sex-related differences in hip and pelvic geometry and abductor mechanics, and adaptation differences between experienced and inexperienced users, may alter routing effects, particularly in the clinical populations of primary interest. This is a primary constraint on the generalizability of our conclusions. Finally, the force profiles were adopted from single-plane hip-assistance optimization studies and kept constant across configurations, which isolates routing geometry only under that fixed timing rather than separating routing geometry from timing in general. Because MT-specific timing could either increase or decrease the observed effects, the present MT results should not be read as a strict lower bound. Future work should therefore optimize force profiles specifically for multiplanar assistance to better reveal its biomechanical potential.

## Conclusion

This study compared SG and MT hip-exosuit cable-routing strategies to evaluate their effects on three-dimensional hip kinematics during treadmill walking. The results showed that MT routing produced phase-dependent kinematic deviations in the hip transverse plane, whereas overall gait-trajectory similarity to unassisted walking remained largely comparable between SG and MT configurations, except in the hip frontal plane during extension. These findings provide preliminary proof-of-concept evidence that routing geometry can be systematically used to modulate transverse-plane hip kinematics, with only limited and indirect evidence of frontal-plane modulation, during assisted walking.

From a clinical standpoint, these findings may offer a useful strategy for selectively addressing rotational gait abnormalities. However, because the present findings were obtained in healthy adults, their direct translation to clinical populations remains uncertain. Individuals with neurological or musculoskeletal gait disorders may respond differently to multiplanar assistance because of impaired sensorimotor function and spasticity. Future work should therefore determine whether similar kinematic effects emerge in patients with rotational gait abnormalities while also incorporating kinetic and muscle-activity outcomes to evaluate clinical relevance more directly.

## Supporting information

S1 File Video abstract: experimental protocol and assisted-condition treadmill walking. Brief methodological overview of the single-actuator soft exosuit and the four cable-routing configurations with representative treadmill walking under the four assisted configurations (FLX-SG, FLX-MT, EXT-SG, EXT-MT). This material corresponds to the treadmill walking trial depicted in Fig 4B. This video published under a CC BY 4.0 license, with written permission from the individual depicted.
(MP4)

S2 Data Processed stride-averaged hip joint angle waveforms across exosuit walking conditions. (XLSX)

S3 Data Dynamic time warping statistics for hip-joint angle waveform comparisons across exosuit walking condition. (XLSX)

S4 Data Peak-value statistics for hip joint-angle changes across exosuit walking condition. (XLSX)

S5 File Within-session measurement repeatability and the size of the significant effects relative to the minimal detectable change. (DOCX)

S6 File Post hoc phase-stratified comparison of kinematic deviations from CON: tabulated Wilcoxon signed-rank results and torque-aligned group-mean joint-angle waveforms for the FLX-MT and EXT-MT conditions. (DOCX)
