# Supplementary material for: Cable-routing geometry as a design variable for multiplanar hip assistance in a single-actuator soft exosuit
Source: PLoS One. 2026 Oct 29;21(10):e0359731. doi: 10.1371/journal.pone.0359731 (PMC13719030; doi:10.1371/journal.pone.0359731)
Supplement: Supplementary file 1 [file pone.0359731.s005.docx]

**Table S5. Within-session measurement repeatability and the size of the significant effects relative to the minimal detectable change.**

**(A) Within-session stride-level repeatability**

| **Plane** | **Stride SD (°)** | **CV (%)** | **ICC(2,1)** | **SEM (°)** | **MDC₉₅ (°)** |
| --- | --- | --- | --- | --- | --- |
| Sagittal | 1.42 | 4.58 | 0.998 | 0.07 | 0.19 |
| Frontal | 0.87 | 7.15 | 0.990 | 0.08 | 0.22 |
| Transverse | 1.13 | 15.82 | 0.944 | 0.25 | 0.70 |

**(B) Significant effects relative to the minimal detectable change**

| **Contrast** | **Plane** | **Effect (°)** | **MDC₉₅ (°)** | **Effect / MDC₉₅** |
| --- | --- | --- | --- | --- |
| FLX-MT vs CON | Sagittal | 3.50 | 0.19 | 18.4 |
| FLX-MT vs FLX-SG | Sagittal | 2.75 | 0.19 | 14.4 |
| EXT-MT vs CON | Sagittal | 2.50 | 0.19 | 13.1 |
| FLX-MT vs FLX-SG | Frontal | 1.20 | 0.22 | 5.4 |
| FLX-MT vs CON | Transverse | 3.60 | 0.70 | 5.1 |
| EXT-MT vs CON | Transverse | 1.80 | 0.70 | 2.6 |
| FLX-SG vs CON | Transverse | 1.10 | 0.70 | 1.6 |

(A) Repeatability metrics were pooled across 10 participants, five conditions, and 10 strides per condition. ICC(2,1) was computed from an odd-versus-even stride split using a two-way random-effects model. SEM was calculated as SD × √(1 − ICC), and MDC₉₅ as 1.96 × √2 × SEM. CV is the coefficient of variation of the stride-to-stride range of motion. (B) Each statistically significant effect reported in the main text is expressed as a multiple of the corresponding MDC₉₅. CON, unassisted control; FLX-SG and FLX-MT, single- and multi-plane flexion assistance; EXT-SG and EXT-MT, single- and multi-plane extension assistance; ICC, intraclass correlation coefficient; SEM, standard error of measurement; MDC₉₅, minimal detectable change at the 95% confidence level; CV, coefficient of variation.
