# Supplementary material for: Cable-routing geometry as a design variable for multiplanar hip assistance in a single-actuator soft exosuit
Source: PLoS One. 2026 Oct 29;21(10):e0359731. doi: 10.1371/journal.pone.0359731 (PMC13719030; doi:10.1371/journal.pone.0359731)
Supplement: Supplementary file 2 [file pone.0359731.s006.docx]

| **Table S6. Torque-phase stratified kinematic deviations from CON.** | | | | | |
| --- | --- | --- | --- | --- | --- |
| **Condition** | **Plane** | **Torque-active** | **Near-zero-torque** | **Ratio  (active / near-zero)** | **p** |
|  |  | **mean \|Δ from CON\| (°)** | |  |  |
| FLX-MT | Sagittal | 5.4 | 2.5 | 2.16 | 0.020 |
| FLX-MT | Transverse | 3.2 | 1.6 | 2.00 | 0.006 |
| EXT-MT | Sagittal | 2.0 | 3.1 | 0.65 | 0.014 |
| EXT-MT | Frontal | 1.2 | 1.9 | 0.63 | 0.020 |
| Deviations are per-subject mean absolute differences between each MT condition and CON, computed at each % of gait cycle and then averaged within each phase before subject-level pairing. Torque-active phases are defined as % of gait cycle where the OpenSim-estimated group-mean assistive torque exceeded 10% of its peak magnitude in the matching plane; near-zero-torque phases are the complement. p-values are from two-tailed Wilcoxon signed-rank tests across the 10 participants, uncorrected. All four contrasts remain significant at α = 0.05. | | | | | |

Fig S6. T****orque-active versus near-zero-torque kinematic deviation from CON, by condition and plane.**** Group-mean hip joint angle waveforms ± 1 SD (n = 10) are plotted for CON (black dashed) and each multi-plane condition (blue solid, FLX-MT; orange solid, EXT-MT), with the OpenSim-estimated group-mean assistive torque overlaid on the right axis (gray dashed) as a descriptive indicator that shares the OpenCap kinematic source with the joint angles. Positive values indicate hip flexion, hip adduction, and hip internal rotation. The yellow band marks the torque-active phase (% of gait cycle where the estimated sagittal-plane group-mean assistive torque exceeded 10% of its peak magnitude); the vertical red dashed line marks the mean toe-off (67% of gait cycle). Annotation boxes report the per-participant mean absolute deviation from CON within each phase and the two-tailed Wilcoxon signed-rank p-value across participants.
